# Impact of Diaphragm-Strengthening Core Training on Postural Stability in High-Intensity Squats

**DOI:** 10.3390/life14121612

**Published:** 2024-12-05

**Authors:** Hyun Seo, Guyeol Jeong, Buongo Chun

**Affiliations:** 1 Department of Sport and Leisure Studies, Graduate School, Korea University, Sejong 02841, Republic of Korea; tjgus456@korea.ac.kr; 2 Department of Physical Education, Chosun University, Gwangju 61452, Republic of Korea; zzzinsang@chosun.ac.kr; 3 Graduate School of Physical Education, Myongji University, Yongin 17058, Republic of Korea

**Keywords:** diaphragm, core training, squat, postural stability, injury prevention

## Abstract

This study analyzed the effects of an 8-week diaphragmatic core training program on postural stability during high-intensity squats and examined its efficacy in injury prevention and performance enhancement. Thirty-seven male participants were randomly assigned to three groups: diaphragmatic core training group (DCTG, n = 12), core training group (CTG, n = 13), and control group (CG, n = 12). Outcome measurements included diaphragm thickness, respiratory function (mean and maximal respiratory pressures), and squat postural stability (distance between the sacral and upper body center points, peak trunk extension moment, peak knee flexion moment, and dynamic postural stability index). Compared to both CTG and CG, DCTG demonstrated significantly greater improvements in diaphragm thickness (DCTG: 34.62% increase vs. CTG: 1.36% and CG: 3.62%, *p* < 0.001), mean respiratory pressure (DCTG: 18.88% vs. CTG: 1.31% and CG: 0.02%, *p* < 0.001), and maximal respiratory pressure (DCTG: 18.62% vs. CTG: 0.72% and CG: 1.90%, *p* < 0.001). DCTG also showed superior improvements in postural stability measures, including reductions in the distance between sacral and upper body center points (DCTG: −6.19% vs. CTG: −3.26% and CG: +4.55%, *p* < 0.05), peak trunk extension moment (DCTG: −15.22% vs. CTG: −5.29% and CG: +19.31%, *p* < 0.001), and dynamic postural stability index (DCTG: −28.13% vs. CTG: −21.43% and CG: no change, *p* < 0.001). No significant between-group differences were observed in peak knee flexion moment. Core training incorporating diaphragmatic strengthening was more effective than conventional training in improving postural stability during high-intensity squats. Core training programs, including diaphragmatic strengthening exercises, may contribute to injury prevention and performance enhancement in exercises requiring lumbar stability, such as squats.

## 1. Introduction

Squats are fundamental weight-training exercises widely popular among athletes and the general population, serving to enhance muscular strength, function, athletic performance, and injury prevention [1,2,3,4]. As a multi-joint exercise, proper kinematic patterns are essential for injury prevention and achieving the intended training objectives [5]. Correct squat execution requires maintenance of a slight thoracic extension with an open chest posture while preserving spinal alignment throughout the movement. Additionally, strong abdominal muscle contraction is necessary to minimize forward trunk leaning and the resulting torque generation [6]. Forward trunk leaning during squats creates torque that deviates from the body’s base of support, increasing the moment arm, and potentially leading to injurious lumbar loading [7].

The inherent structural instability of the lumbar spine necessitates mechanical spinal rigidity. Core muscles, including the obliques, rectus abdominals, transversus abdominis, multifidus spine, and diaphragm, generate substantial intra-abdominal pressure (IAP) that increases spinal segmental stiffness, preventing excessive movement and enhancing spinal stability [8,9,10,11]. The increased IAP from the strengthened core musculature counteracts improper movement patterns during squats while enhancing muscular coordination, thereby improving dynamic stability and reducing injury risk [12,13].

The diaphragm plays an important role in generating and maintaining strong IAP during squatting. Beyond its primary respiratory function, which accounts for approximately 70% of breathing capacity, diaphragmatic contraction regulates abdominal content displacement and pressurization. The resulting elevated IAP increases thoracolumbar fascial tension through eccentric contraction of the transversus abdominis [14,15]. This mechanism contributes to lumbar stabilization through tension in the erector spinae and multifidus muscles attached to the posterior thoracolumbar fascia as well as the external obliques attached to its lateral borders. Furthermore, activated multifidus muscles originating from the sacrum enhance sacroiliac joint stability, thereby improving pelvic stability [16].

The diaphragm functions not only as a respiratory muscle but also as a core stabilizer, working synergistically with other core muscles [17]. It activates the trunk musculature and maintains proper segmental alignment, demonstrating its close relationship with postural control [18]. The increased IAP resulting from diaphragmatic strengthening enables stable movement initiation through spinal stabilization [19], suggesting its crucial role in maintaining stability and reducing injury risk during heavy load exercises such as squats.

Recent research on squat-related injury prevention has focused on various aspects including prime mover muscle activation studies [20,21,22], comparative analyses of different squat techniques (front versus back squats) (Warneke et al. [23]; Junior et al. [24]), and investigations of stance width and depth variations [25,26,27]. While these studies provide valuable insights into squat execution and methodology, there remains a notable gap in research examining the relationship between diaphragmatic strengthening and squat posture stability, despite the significant contribution of the diaphragm to spinal stability among the core muscles.

Therefore, this study aimed to scientifically validate the effectiveness of diaphragmatic strengthening, a primary contributor to IAP formation and trunk stability, in improving postural stability during squatting, with implications for injury prevention and performance enhancement.

## 2. Materials and Methods

### 2.1. Participants

Male participants in their twenties who voluntarily agreed to participate were recruited for this study. The inclusion criteria were at least 1 year of regular weight-training experience and the ability to perform five or more squats at 80% of their one rep max. The exclusion criterion was musculoskeletal or respiratory disorders within the previous 6 months.

Prior to the commencement of the study, all selected participants were thoroughly briefed on the study’s purpose and procedures, and informed consent was obtained for data collection. Initially, 39 participants were recruited, however, 2 withdrew during the study, resulting in a final sample of 37 participants.

### 2.2. Research Design

**Figure 1:**
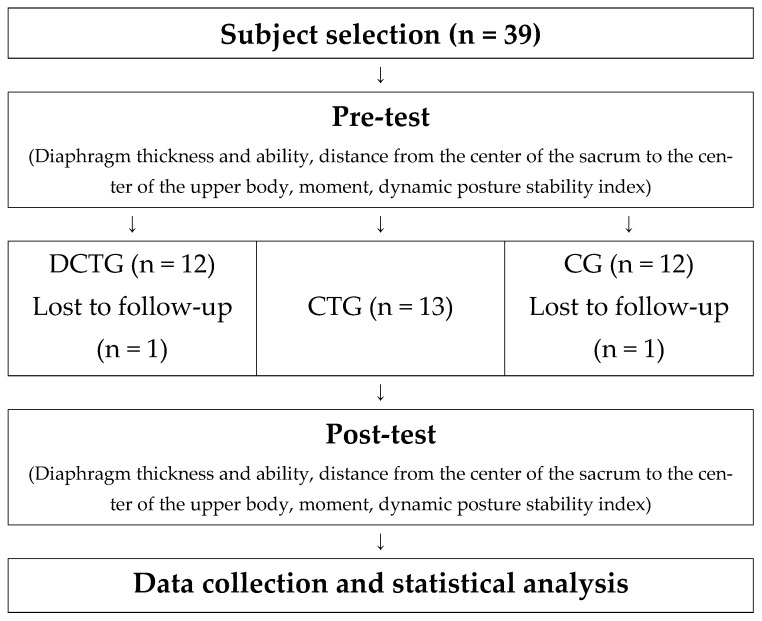
Study process.

The participants were randomly assigned to three groups using a lottery method to ensure objectivity and reliability. The first group, designated the diaphragm strengthening and core training group (DCTG), received both diaphragmatic strengthening exercises and core training. The second group, the core training group (CTG), performed only core training. The third group served as the control group (CG) and did not receive any intervention. This design was implemented to compare the effectiveness of the training methods and specifically to evaluate the additional impact of diaphragmatic strengthening on core training (Figure 1). Participants’ demographic characteristics are shown in Table 1.

### 2.3. Experimental Procedures

All participants underwent measurements before and after the intervention period under identical laboratory conditions. The diaphragmatic exercise program was conducted twice daily (morning and afternoon) for 6 days per week, with each session lasting up to 5 min. The core training program consisted of 50 min sessions conducted thrice per week for 8 weeks. The measured variables included diaphragm thickness and function for diaphragmatic strengthening assessment and squat postural stability variables, including the distance from the sacral center to the upper body center, peak trunk extension moment, peak knee flexion moment, and dynamic postural stability index (DPSI).

### 2.4. Measurement Methods

#### 2.4.1. Diaphragm Thickness Measurement

**Figure 2:**
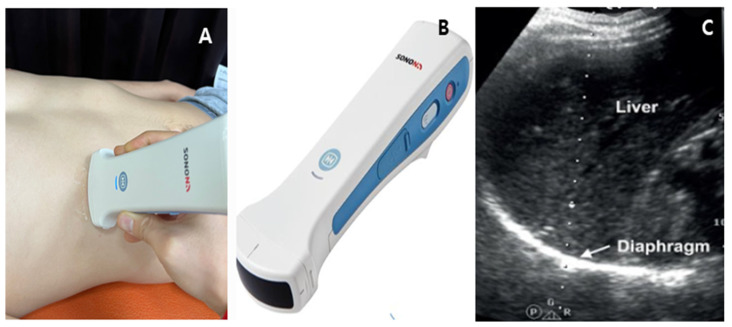
Measurement of Diaphragm Thickness. (**A**) The transducer is positioned on the intercostal space in the anterior axillary line. (**B**) Diaphragm measurement equipment (SONON Convex 300c, Healcerion, Seoul, Republic of Korea). (**C**) Two-dimensional image of the diaphragm.

Diaphragmatic thickness was measured using a SONON convex 300c ultrasound probe (Healcerion, Seoul, Republic of Korea) (Figure 2). The participants laid in the supine position with their knees flexed at 90°. The probe was positioned between the 7th and 8th or 8th and 9th intercostal spaces, where the diaphragm muscle was attached. Two-dimensional imaging was used to measure the distance between the pleural and peritoneal lines, and the average of three measurements was recorded [28,29].

#### 2.4.2. Diaphragm Function Assessment

**Figure 3:**
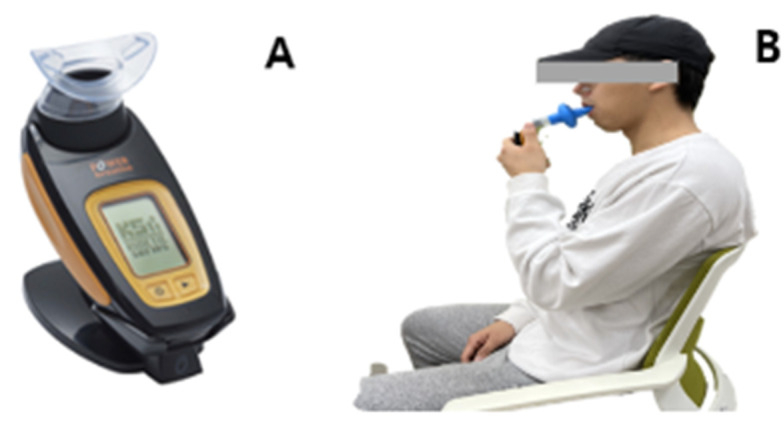
Measurement of Diaphragm Function (**A**) Power Breathe K5 (POWERbreathe International Ltd., Southam, Warwickshire, UK), (**B**) Diaphragm function measurement posture.

Diaphragm function was evaluated using Power Breathe K5 (POWERbreathe International Ltd., Southam, Warwickshire, UK). The participants sat comfortably with their backs supported and noses occluded, and performed 30 maximal inspiratory efforts using a disposable mouthpiece. Average inspiratory pressure and peak inspiratory pressure were recorded [30] (Figure 3).

#### 2.4.3. Squat Motion Analysis

Motion analysis was conducted using 10 Raptor-E infrared cameras (Motion Analysis) at 120 frames/s. Nonlinear transformation was used for three-dimensional spatial coordinate calibration. The Helen–Hayes marker set [31] was applied with 24 reflective markers to define joint and segment coordinates. Data were analyzed using the Cortex software.

#### 2.4.4. Dynamic Postural Stability Assessment

Dynamic postural stability was measured during squatting using force plates. Ground reaction force data were used to calculate the mediolateral stability index (MLSI), anteroposterior stability index (APSI), vertical stability index (VSI), and DPSI [32]. The DPSI is calculated as follows:

$$MLSI=\sqrt{\sum (0-{x)}^{2}/\;number\;of\;data\;poings}$$

$$APSI=\sqrt{\sum (0-{y)}^{2}/\;number\;of\;data\;poings}$$

$$VSI=\sqrt{\sum (body\;weight-{z)}^{2}/\;number\;of\;data\;poings}$$

DPSI = MLSI +APSI +VSI

MLSI, medial lateral stability index; APSI, anterior posterior stability index; VSI, vertical stability index; DPSI, dynamic postural stability index.

#### 2.4.5. Squat Testing Protocol

Squat motion and dynamic postural stability measurements were conducted during five repetitions at 80% of 1-RM. Parallel squat depth was selected as the standardized position for all measurements, defined as the position where the thigh is parallel to the ground with approximately 90-degree knee flexion. This position was chosen to ensure measurement consistency across participants while maintaining practical relevance to common training applications. One-repetition maximum (1-RM) was determined one week prior to the main testing sessions following the National Strength and Conditioning Association (NSCA) guidelines using indirect measurement methods. The 1-RM assessment protocol consisted of progressive loading: 50% of estimated 1-RM for 10 repetitions, 70% for 5 repetitions, 80% for 3 repetitions, and 90% for 1 repetition, with 3 min rest intervals between sets. The warm-up protocol for the main testing sessions included 5 min of light cycling, dynamic stretching, and progressive loading sets (40%, 60% of 1-RM) before the measured sets at 80% 1-RM. During the testing sets, participants performed 5 repetitions with controlled tempo (2-s eccentric, 1-s pause, 2-s concentric phases), with 3-min rest intervals between sets to minimize fatigue effects.

### 2.5. Exercise Programs

#### 2.5.1. Diaphragmatic Training

Diaphragmatic training uses the Power Breathe Plus device, which provides variable resistance through color-coded spring-loaded valves. Following the protocols established by Griffiths and McConnell [33] and Romer and McConnell [34], participants performed 30 repetitions twice daily (morning and afternoon) for six days per week. Each session consisted of 30 maximal inspiratory efforts. The initial training intensity was set to a resistance level allowing 30 repetitions at maximal effort (30RM). The participants self-adjusted their intensity periodically when they could complete all repetitions with proper form [34], with sessions limited to 5 min [35]. This training duration and frequency were selected based on the diaphragm’s physiological characteristics, particularly its composition of approximately 55% slow-twitch fibers, which responds effectively to moderate-load, high-repetition training protocols [33]. The twice-daily sessions with adequate rest intervals were designed to optimize diaphragmatic adaptations while preventing respiratory muscle fatigue, as supported by previous respiratory muscle training research [34,35].

#### 2.5.2. Core Training

The 8-week core training program consisted of three weekly sessions, each comprising 5 min of warm-up, 40 min of the main exercise, and 5 min of cool-down. Exercise intensity was set at moderate levels (RPE 12–15) [36,37], following the American College of Sports Medicine [38] guidelines and NASM core training principles [39] (Table 2).

### 2.6. Statistical Analysis

Data were analyzed using Statistical Package for Social Sciences version 26.0 (IBM Corp., Armonk, NY, USA). The mean and standard deviation were calculated for all variables. Analysis of covariance (ANCOVA) was performed using pre-test values as covariates to determine between-group differences, with least significant difference post hoc tests for the comparison of main effects. Statistical significance was set at α = 0.05.

## 3. Results

### 3.1. Effects of Diaphragm-Strengthening Core Training on Diaphragm Thickness and Function

Analysis of diaphragm thickness revealed significant changes across the groups. DCTG showed a significant increase of 34.62% from 2.34 ± 0.33 to 3.15 ± 0.41 mm, CTG showed a 1.36% increase from 2.20 ± 0.26 to 2.23 ± 0.25 mm, and CG showed a 3.62% increase from 2.21 ± 0.21 to 2.29 ± 0.32 mm (Table 3). ANCOVA revealed significant between-group differences (F = 42.032, *p* < 0.001). Post hoc analysis indicated that DCTG exhibited significantly greater increases in diaphragm thickness than CTG and CG (*p* < 0.05).

Average respiratory pressure analysis showed an 18.88% increase in DCTG from 94.71 ± 22.01 to 112.59 ± 19.01 cmH_2_O, 1.31% increase in CTG from 87.95 ± 16.66 to 89.10 ± 13.48 cmH_2_O, and a minimal 0.02% increase in CG from 98.81 ± 18.68 to 98.87 ± 2.23 cmH_2_O (Table 3). ANCOVA revealed significant between-group differences (F = 21.613, *p* < 0.001). Post hoc analysis indicated that DCTG showed significantly greater improvements in average respiratory pressure than CG (*p* < 0.05).

Maximum respiratory pressure analysis revealed an 18.62% increase in DCTG from 107.77 ± 24.29 to 127.84 ± 19.96 cmH_2_O, 0.72% increase in CTG from 117.96 ± 11.43 to 118.81 ± 11.51 cmH_2_O, and 1.90% increase in CG from 119.20 ± 14.77 to 121.47 ± 16.60 cmH_2_O (Table 3). ANCOVA indicated significant between-group differences (F = 19.919, *p* < 0.001). Post hoc analysis revealed that DCTG exhibited significantly greater improvements in maximum respiratory pressure than CG (*p* < 0.05).

### 3.2. Changes in Squat Posture Following Diaphragm-Strengthening Core Training

**Figure 4:**
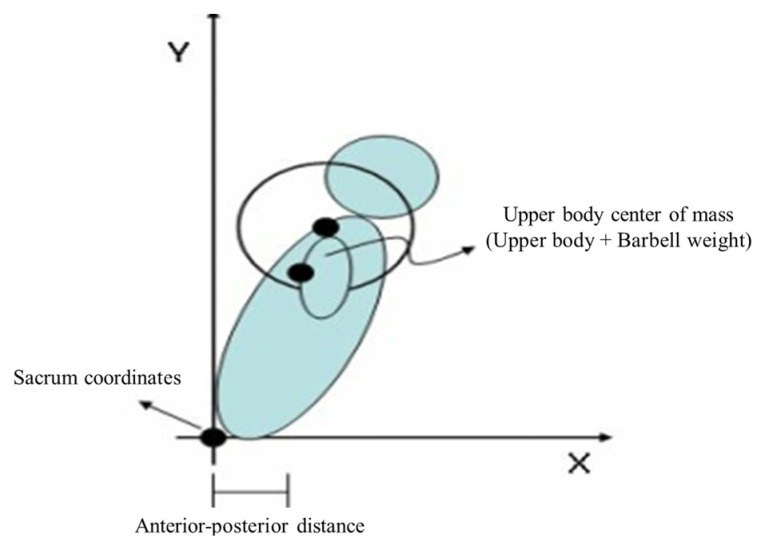
Distance measurement between the sacral center and upper body center points during squat performance.

Postural changes during squats were analyzed at the deepest point of movement, where the thighs were parallel to the ground. This position typically corresponded to the maximum distance between the sacral center point and the upper body center point. The analysis included comparisons of the peak trunk extension and knee flexion moments relative to this distance (Figure 4).

Analysis of the distance between the sacral and upper body center points revealed that DCTG showed a 6.19% decrease from 26.32 ± 2.28 to 24.69 ± 2.71 cm, CTG showed a 3.26% decrease from 26.11 ± 2.21 to 25.26 ± 1.47 cm, and CG showed a 4.55% increase from 24.20 ± 2.05 to 25.30 ± 1.16 cm (Table 4). ANCOVA revealed significant between-group differences (F = 3.989, *p* < 0.05). Post hoc analysis indicated that DCTG exhibited a significantly greater reduction in this distance than CG (*p* < 0.05).

Peak trunk extension moment analysis showed a 15.22% decrease in DCTG from 1.84 ± 0.1 to 1.56 ± 0.14 N·m and a 5.29% decrease in CTG from 1.70 ± 0.24 to 1.61 ± 0.15 N·m. In contrast, CG showed a 19.31% increase from 1.45 ± 0.21 to 1.73 ± 0.31 N·m (Table 4). ANCOVA revealed significant between-group differences (F = 3.516, *p* < 0.05). Post hoc analysis indicated that both DCTG and CTG demonstrated significantly greater reductions in peak trunk extension moment than CG (*p* < 0.05).

Analysis of the peak knee flexion moment showed a 1.44% increase in DCTG from −1.39 ± 0.27 to −1.41 ± 0.24 N·m and a 6.15% increase in CTG from −1.30 ± 0.25 to −1.38 ± 0.28 N·m, while CG showed no change (Table 4). ANCOVA revealed no significant between-group differences (F = 1.066, *p* > 0.05), and post hoc analysis showed no significant differences among the three groups.

DPSI analysis demonstrated a 28.13% decrease in DCTG from 0.32 ± 0.63 to 0.23 ± 0.05 and a 21.43% decrease in CTG from 0.28 ± 0.72 to 0.22 ± 0.08, while CG showed no change (Table 4). ANCOVA revealed significant between-group differences (F = 3.401, *p* < 0.05). Post hoc analysis indicated that DCTG showed a significantly greater reduction in the DPSI than CTG and CG (*p* < 0.05).

## 4. Discussion

This study aimed to scientifically evaluate the effects of an 8-week diaphragmatic core training program on postural stability during high-intensity squats, with a specific focus on injury prevention and performance enhancement. Unlike previous core training studies, the originality of this study lies in its focus on diaphragmatic strengthening. The results demonstrated that diaphragmatic core training, compared with conventional core training, produced significant improvements in diaphragm thickness, average/maximum respiratory pressure, sacral-to-upper body center point distance, peak trunk extension moment, and DPSI. Notably, increased diaphragmatic thickness contributes to enhanced respiratory function and spinal stability, which are crucial for efficient squat performance and injury prevention.

### 4.1. Changes in Diaphragm Thickness and Respiratory Function

The diaphragm, the primary inspiratory muscle, is essential for maintaining respiratory functions and life [40]. Kraemer et al. [41] reported that, similarly to other skeletal muscles, the diaphragm undergoes hypertrophy when subjected to appropriate exercise loads. Our findings suggest that the applied physiological load was sufficient to induce diaphragmatic hypertrophy, which is consistent with Enright et al.’s [42] study, demonstrating that inspiratory muscle training can promote structural changes and strength improvements in the diaphragm. Furthermore, diaphragmatic hypertrophy, which leads to increased muscle cross-sectional area [43], appears to enhance the activation of the diaphragm and auxiliary muscles during inspiration, resulting in improved average and maximum respiratory pressures.

The physiological basis for these adaptations lies in the diaphragm’s unique muscle fiber composition, consisting of 55–65% Type I fibers with high oxidative capacity [44]. This skeletal muscle characteristic allows the diaphragm to respond effectively to respiratory muscle training principles, similarly to other skeletal muscles [45]. Previous research has demonstrated that targeted respiratory and inspiratory muscle training can enhance diaphragmatic thickness, inspiratory capacity, and exercise performance [46]. The resistance loading protocol specifically targeting the diaphragm as the primary inspiratory muscle [33,47] proved effective in eliciting these adaptations.

These findings extend previous research on respiratory muscle training interventions. While traditional core training studies have primarily focused on abdominal and back muscle activation [48,49], the present results demonstrate the additional benefits of incorporating specific diaphragmatic strengthening. Our findings align with previous respiratory training studies in showing significant improvements in respiratory pressures. For example, HajGhanbari et al. [50] reported improvements in inspiratory muscle strength through conventional inspiratory muscle training, while Seixas et al. [51] demonstrated enhanced respiratory function through pressure threshold loading. However, the current study uniquely demonstrates that these respiratory improvements can be achieved while simultaneously enhancing postural stability during complex movements like squats. This dual benefit suggests that integrating diaphragmatic training with core exercises may be more efficient than addressing these aspects separately.

Downey et al. [52] reported a 24.5% increase in inspiratory muscle strength following diaphragmatic training, corroborating numerous studies demonstrating structural changes and improved inspiratory strength through respiratory muscle training [34,42,47,53,54].

Although Kraemer et al. [55] recommended 80% intensity for inspiratory muscle strengthening, our study employed 50% intensity based on Griffiths and McConnell’s [33] methodology, considering the diaphragm fiber composition (55% slow-twitch fibers). This aligns with recent research showing comparable effects of low-intensity/high-repetition and high-intensity/low-repetition training on muscle hypertrophy and strength gain [56,57,58].

### 4.2. Changes in Postural Stability During High-Intensity Squats

The diaphragmatic core training group demonstrated significant reductions in sacral-to-upper body center point distance and peak trunk extension moment compared with the other groups, while knee flexion moment showed no significant between-group differences. The reduced distance between the sacral and upper body center points suggests improved spinal stability through increased IAP [59], which is consistent with previous research indicating that minimizing the distance between the body centerline and barbell promotes more efficient squat performance [60].

The non-significant differences in maximum knee flexion moment between groups may be attributed to several biomechanical factors. The trend toward increased maximum knee flexion moment in both training groups likely reflects a postural adaptation. Specifically, as participants achieved improved trunk stability and reduced forward lean, a compensatory increase in knee anterior displacement occurred to maintain balance during the squat. This mechanical relationship between trunk position and knee moment aligns with previous research [3]. While the training programs effectively enhanced trunk stability, they may not have directly influenced knee joint mechanics, as knee moment is also affected by individual factors such as squat technique and lower limb mobility. These findings emphasize the importance of considering both spinal and knee joint biomechanics in squat performance.

### 4.3. Changes in Dynamic Stability

The significant reduction in the DPSI in the diaphragmatic core training group indicated improved overall movement control. The DPSI, which incorporates anterior–posterior, medial–lateral, and vertical stability components [32], showed that participants maintained their center of mass more effectively within the base of support, suggesting enhanced adaptability to external forces and improved balance control [61].

These improvements in postural stability can be explained by the physiological mechanisms of diaphragmatic function. Strengthened diaphragmatic function increases intra-abdominal pressure, which enhances spinal stability by restricting vertebral movement [62]. This increased pressure also enhances tension in the thoracolumbar fascia, a connective tissue structure linking various trunk muscles including the transversus abdominis, multifidus, and erector spinae [63]. This integrated system of increased intra-abdominal pressure and fascial tension improves both spinal segmental stability and sacroiliac joint function [64], explaining the enhanced postural stability observed during high-intensity squats.

Studies analyzing foot pressure during squat performance [65], examining moment analysis relative to trunk angle during squats [60,66], and demonstrating the positive effects of weight belts during squat exercises [67,68] support our findings. Furthermore, Gholami et al. [69] observed reduced ankle movement and increased lumbar stabilization after respiratory training in patients with low back pain. These findings suggest that maintaining stability requires maintaining the center of mass within the base of the support to reduce the moment arm length and decrease lumbar extension torque, thereby reducing spinal loading [70]. Therefore, diaphragmatic core training appears to enhance dynamic postural stability, potentially reducing unnecessary movements during squat performance and contributing to injury prevention.

## 5. Conclusions

Core training demonstrated positive effects on squat posture and dynamic postural stability, with diaphragmatic strengthening combined with core training proving more effective than conventional core training alone. Specifically, significant improvements were observed in diaphragmatic thickness and function, reduction in sacral-to-upper body center point distance, decreased peak trunk extension moment, and enhanced DPSI. These findings suggest that incorporating diaphragmatic strengthening exercises into core training programs effectively prevents injuries and enhances performance during exercises that require lumbar stability, such as squats. Therefore, diaphragmatic strengthening exercises are recommended in weight training programs for both athletes and recreational exercisers.

## 6. Study Limitations

The study examined the effects of diaphragmatic strengthening on postural stability mechanisms during high-intensity squats. Postural stability represents one of several factors contributing to injury prevention during squats, alongside muscle strength imbalances, flexibility, and technical execution. The findings are limited to male participants, preventing generalization to female populations due to potential differences in neuromuscular control patterns, hormonal influences, and anatomical characteristics affecting stability mechanisms. The study design included only pre- and post-intervention measurements over the 8-week period, limiting the identification of temporal progression in stability adaptations. Additionally, the 8-week intervention period only revealed immediate training effects. Long-term follow-up studies are needed to determine whether these improvements in stability and respiratory function are maintained and translate to sustained injury prevention benefits. Further investigation with various measurement intervals is needed to determine the optimal duration and progression of diaphragmatic strengthening effects. The results demonstrated improvements in stability parameters at 80% 1-RM; however, additional research should explore the relationship between enhanced stability and squat performance metrics, including strength development and technical efficiency under various loading conditions. While the core training group served as an active control for diaphragmatic training effects, the control group design without any intervention limited the ability to control for potential placebo effects. Future research designs should include control group activities matched for time and effort expenditure, such as light physical activity or stretching, to better isolate the specific effects of diaphragmatic training.

## Figures and Tables

**Table 1:** Participant characteristics.

Group	Age (yrs)	Height (cm)	Weight (kg)
DCGT (n = 12)	21.75 ± 3.04	176.97 ± 7.48	75.13 ± 8.43
CTG (n = 13)	21.23 ± 1.58	176.36 ± 7.27	74.55 ± 7.33
CG (n = 12)	22.08 ± 1.67	176.33 ± 5.84	76.68 ± 8.87
Sig	0.622	0.967	0.803

**Table 2:** Core training program.

Workout Types		Set	Rest	Intensity
Warm up (5 min)	Stretching			RPE 9
Main exercise (40 min)	Hip bridge, Plank Side Plank, crunches oblique crunches Dead bug (statics, dynamics) Bird dog (statics, dynamics)	4 set	60 sec	RPE 12–15
	Side Plank Rotation, Plank single-leg tuck Plank shoulder taps, Crawling Dead bug (one arm & one leg down) Bird dog (one arm & one leg down)			
Cool down (5 min)	Stretching			RPE 9

**Table 3:** Changes in diaphragm thickness and function following diaphragm-strengthening core training.

Group	Pre (M ± SD)	Post (M ± SD)	Correction (M ± SD)	∆%	*p*-Value	Post-Hoc	ANCOVA				
							SS	DF	MS	F-Value	*p*
**The change of diaphragm thickness (Unit: mm)**											
DCTG (n = 12)	2.34 ± 0.33	3.15 ± 0.41	3.06 ± 0.06	34.62	0.000 ***	a > b, c	2.145	1	2.145	40.767	0.000 ***
CTG (n = 13)	2.20 ± 0.26	2.23 ± 0.25	2.27 ± 0.06	1.36	0.281		4.424	2	2.212	42.032	0.000 ***
CG (n = 12)	2.21 ± 0.21	2.29 ± 0.32	2.33 ± 0.06	3.62	0.209		1.737	33	0.053		
**The change of diaphragm average respiratory pressure (Unit: cmH_2_O)**											
DCTG (n = 12)	94.71 ± 22.01	112.59 ± 19.01	111.79 ± 2.20	18.88	0.000 ***	a > b, c	6997	1	6997	119.871	0.000 ***
CTG (n = 13)	87.95 ± 16.66	89.10 ± 13.48	93.50 ± 2.15	1.31	0.618		2523.114	2	1261.557	21.613	0.000 ***
CG (n = 12)	98.81 ± 18.68	98.83 ± 15.85	94.87 ± 2.23	0.02	0.987		1926.239	33	58.371		
**The change of diaphragm maximum respiratory pressure (Unit: cmH_2_O)**											
DCTG (n = 12)	107.77 ± 24.29	127.84 ± 19.96	133.91 ± 2.16	18.62	0.000 ***	a > b, c	7265.84	1	7265.84	137.538	0.000 ***
CTG (n = 13)	117.96 ± 11.43	118.81 ± 11.51	116.39 ± 2.02	0.72	0.597		2104.511	2	1052.256	19.919	0.000 ***
CG (n = 12)	119.20 ± 14.77	121.47 ± 16.60	118.02 ± 2.11	1.9	0.132		1743.315	33	52.828		

Notes: Values are presented as mean ± standard deviation. *** *p* < 0.001 DCTG: Diaphragm strengthening & Core Training Group (a) CTG: Core Training Group (b) CG: Control Group (c) ∆% = [(Post − Pre)/Pre] × 100 Post hoc significance: a > b, c indicates DCTG showed significantly greater improvements compared to both CTG and CG.

**Table 4:** Changes in squat posture parameters following diaphragm-strengthening core training.

Group	Pre (M ± SD)	Post (M ± SD)	Correction (M ± SD)	∆%	*p*-Value	Post-Hoc	ANCOVA				
							SS	DF	MS	*F*	*p*
**The change of distance between the center of the sacrum and the center of the upper body (unit: cm)**											
DCTG (n = 12)	26.32 ± 2.28	24.69 ± 2.71	24.26 ± 0.42	−6.19	0.004 **	a > c	52.289	1	52.289	24.695	0.000 ***
CTG (n = 13)	26.11 ± 2.21	25.26 ± 1.47	24.95 ± 0.40	−3.26	0.153		16.892	2	8.446	3.989	0.028 **
CG (n = 12)	24.20 ± 2.05	25.30 ± 1.16	26.07 ± 0.44	4.55	0.029 *		69.873	33	2.117		
**The change of maximum trunk extension moment (unit: N·m)**											
DCTG (n = 12)	1.84 ± 0.10	1.56 ± 0.14	1.51 ± 0.06	−15.22	0.000 ***	a > c, b > c	0.127	1	0.127	2.853	0.000 ***
CTG (n = 13)	1.70 ± 0.24	1.61 ± 0.15	1.60 ± 0.59	−5.29	0.258		0.313	2	0.157	3.516	0.041 *
CG (n = 12)	1.45 ± 0.21	1.73 ± 0.31	1.80 ± 0.72	19.31	0.005 **		1.469	33	0.045		
**The change of maximum knee flexion moment (unit: N·m)**											
DCTG (n = 12)	−1.39 ± 0.27	−1.41 ± 0.24	−1.35 ± 0.04	1.44	0.764	n/a	1.079	1	1.079	41.829	0.000 ***
CTG (n = 13)	−1.30 ± 0.25	−1.38 ± 0.28	−1.40 ± 0.04	6.15	0.189		0.055	2	0.027	1.066	0.346
CG (n = 12)	−1.26 ± 0.17	−1.26 ± 0.16	−1.30 ± 0.04	0	0.891		0.851	33	0.026		
**The change of dynamic postural stability index**											
DCTG (n = 12)	0.32 ± 0.63	0.23 ± 0.05	0.21 ± 0.01	−28.13	0.000 ***	a > b > c	0.057	1	0.057	15.208	0.000 ***
CTG (n = 13)	0.28 ± 0.72	0.22 ± 0.08	0.23 ± 0.01	−21.43	0.054		0.026	2	0.013	3.401	0.045 *
CG (n = 12)	0.26 ± 0.76	0.26 ± 0.08	0.28 ± 0.01	0	0.71		0.124	33	0.004		

Notes: Values are presented as mean ± standard deviation. * *p* < 0.05, ** *p* < 0.01, *** *p* < 0.001 DCTG: Diaphragm strengthening & Core Training Group (a) CTG: Core Training Group (b) CG: Control Group (c) ∆% = [(Post-Pre)/Pre] × 100 Post hoc significance: a, b, c indicates DCTG showed significantly greater improvements compared to both CTG and CG.

## Data Availability

The data that support the findings of this study are available from the corresponding author (H.S.) upon reasonable request.

## References

[1] Sprague E., Reynolds S., Brindley P. (2016). Patient isolation precautions: Are they worth it?. Can. Respir. J..

[2] Escamilla R.F., Fleisig G.S., Zheng N., Lander J.E., Barrentine S.W., Andrews J.R., Bergemann B.W., Moorman C.T. (2001). Effects of technique variations on knee biomechanics during the squat and leg press. Med. Sci. Sports Exerc..

[3] Fry A.C., Smith J.C., Schilling B.K. (2003). Effects of knee position on hip and knee torques during the barbell squat. J. Strength Cond. Res..

[4] McCurdy K.W., Langford G.A., Doscher M.W., Wiley L.P., Mallard K.G. (2005). The effects of short-term unilateral and bilateral lower-body resistance training on measures of strength and power. J. Strength Cond. Res..

[5] Králik S., Freudenfeld D., Gurín D. (2021). Changes in the activity of selected muscles during deep squat correction. Slovak. J. Sports Sci..

[6] Donnelly D.V., Berg W.P., Fiske D.M. (2006). The effect of the direction of gaze on the kinematics of the squat exercise. J. Strength Cond. Res..

[7] Williams K.R. (1980). Biomechanical factors contributing to injury during trunk loading. Ergonomics.

[8] Stokes I.A., Gardner-Morse M., Henry S.M., Badger G.J. (2000). Decrease in trunk muscular response to perturbation with preactivation of lumbar spinal musculature. Spine.

[9] Franklin T.C., Granata K.P. (2007). Role of reflex gain and reflex delay in spinal stability—A dynamic simulation. J. Biomech..

[10] Cholewicki J., Ivancic P.C., Radebold A. (2002). Can increased intra-abdominal pressure in humans be decoupled from trunk muscle co-contraction during steady state isometric exertions?. Eur. J. Appl. Physiol..

[11] Lee P.J., Rogers E.L., Granata K.P. (2006). Active trunk stiffness increases with co-contraction. J. Electromyogr. Kinesiol..

[12] Ford K.R., Myer G.D., Hewett T.E. (2007). Increased trunk motion in female athletes compared to males during single leg landing. Med. Sci. Sports Exerc..

[13] Myer G.D., Chu D.A., Brent J.L., Hewett T.E. (2008). Trunk and hip control neuromuscular training for the prevention of knee joint injury. Clin. Sports Med..

[14] Essendrop M., Andersen T.B., Schibye B. (2002). Increase in spinal stability obtained at levels of intra-abdominal pressure and back muscle activity realistic to work situations. Appl. Ergon..

[15] Baechle T., Earle W. (2008). Essentials of Strength Training and Conditioning.

[16] Barker P.J., Briggs C.A., Bogeski G. (2004). Tensile transmission across the lumbar fasciae in unembalmed cadavers: Effects of tension to various muscular attachments. Spine.

[17] Janssens L., McConnell A.K., Pijnenburg M., Claeys K., Goossens N., Lysens R., Troosters T., Brumagne S. (2015). Inspiratory muscle training affects proprioceptive use and low back pain. Med. Sci. Sports Exerc..

[18] Hodges P.W., Moseley G.L., Gabrielsson A., Gandevia S.C. (2003). Experimental muscle pain changes feedforward postural responses of the trunk muscles. Exp. Brain Res..

[19] Cholewicki J., Juluru K., McGill S.M. (1999). Intra-abdominal pressure mechanism for stabilizing the lumbar spine. J. Biomech..

[20] Williams M.J., Gibson N.V., Sorbie G.G., Ugbolue U.C., Brouner J., Easton C. (2021). Activation of the gluteus maximus during performance of the back squat, split squat, and barbell hip thrust and the relationship with maximal sprinting. J. Strength Cond. Res..

[21] Vantrease W.C., Townsend J.R., Sapp P.A., Henry R.N., Johnson K.D. (2021). Maximal strength, muscle activation, and bar velocity comparisons between squatting with a traditional or safety squat bar. J. Strength Cond. Res..

[22] Coratella G., Tornatore G., Caccavale F., Longo S., Esposito F., Cè E. (2021). The activation of gluteal, thigh, and lower back muscles in different squat variations performed by competitive bodybuilders: Implications for resistance training. Int. J. Environ. Res. Public Health.

[23] Warneke K., Keiner M., Schiemann S., Lohmann L., Wirth K. (2023). Influence of maximal strength performance in front squat and deadlift on linear sprint and jump performance in male youth elite basketball players. Ger. J. Exerc. Sport Res..

[24] Junior G.D.B.V., Passos R.P., Júnior J.R.G., dos Santos Carvalho A., Abdalla P.P. (2022). Squat with front and back bar position in parallel and sumo feet techniques: A cross-sectional study. Rev. CPAQV.

[25] Barrett K.B., Sievert Z.A., Bennett H.J. (2023). A comparison of squat depth and sex on knee kinematics and muscle activation. J. Biomech. Eng..

[26] Chan C.K., Azah H.N., Yeow C.H., Goh S.K., Ting H.N., Salmah K. (2022). Effects of squatting speed and depth on lower extremity kinematics, kinetics and energetics. J. Mech. Med. Biol..

[27] Sinclair J., Taylor P.J., Jones B., Butters B., Bentley I., Edmundson C.J. (2022). A multi-experiment investigation of the effects stance width on the biomechanics of the barbell squat. Sports.

[28] Boon A.J., Harper C.J., Ghahfarokhi L.S., Strommen J.A., Watson J.C., Sorenson E.J. (2013). Two-dimensional ultrasound imaging of the diaphragm: Quantitative values in normal subjects. Muscle Nerve.

[29] Hellyer N.J., Andreas N.M., Bernstetter A.S., Cieslak K.R., Donahue G.F., Steiner E.A., Hollman J.H., Boon A.J. (2017). Comparison of diaphragm thickness measurements among postures via ultrasound imaging. PM R.

[30] Minahan C., Sheehan B., Doutreband R., Kirkwood T., Reeves D., Cross T. (2015). Repeated-sprint cycling does not induce respiratory muscle fatigue in active adults: Measurements from the powerbreathe^®^ inspiratory muscle trainer. J. Sports Sci. Med..

[31] Kadaba M.P., Ramakrishnan H.K., Wootten M.E. (1990). Measurement of lower extremity kinematics during level walking. J. Orthop. Res..

[32] Wikstrom E.A., Tillman M.D., Schenker S.M., Borsa P.A. (2008). Jump-landing direction influences dynamic postural stability scores. J. Sci. Med. Sport.

[33] Griffiths L.A., McConnell A.K. (2007). The influence of inspiratory and expiratory muscle training upon rowing performance. Eur. J. Appl. Physiol..

[34] Romer L.M., McConnell A.K., Jones D.A. (2002). Inspiratory muscle fatigue in trained cyclists: Effects of inspiratory muscle training. Med. Sci. Sports Exerc..

[35] Klusiewicz A., Borkowski L., Zdanowicz R., Boros P., Wesołowski S. (2008). The inspiratory muscle training in elite rowers. J. Sports Med. Phys. Fitness.

[36] Cosio-Lima L.M., Reynolds K.L., Winter C., Paolone V., Jones M.T. (2003). Effects of physioball and conventional floor exercises on early phase adaptations in back and abdominal core stability and balance in women. J. Strength Cond. Res..

[37] Mills J.D., Taunton J.E., Mills W.A. (2005). The effect of a 10-week training regimen on lumbo-pelvic stability and athletic performance in female athletes: A randomized-controlled trial. Phys. Ther. Sport.

[38] American College of Sports Medicine (ACSM) (2012). ACSM’s Guidelines for Exercise Testing and Prescription.

[39] Clark M.A., Lucett S., Corn R.J. (2008). NASM Essentials of Personal Fitness Training.

[40] Levitzky M.G. (2007). Pulmonary Physiology.

[41] Kraemer W.J., Fleck S.J., Evans W.J. (1996). Strength and power training: Physiological mechanisms of adaptation. Exerc. Sport Sci. Rev..

[42] Enright S.J., Unnithan V.B., Heward C., Withnall L., Davies D.H. (2006). Effect of high-intensity inspiratory muscle training on lung volumes, diaphragm thickness, and exercise capacity in subjects who are healthy. Phys. Ther..

[43] Helms E.R., Byrnes R.K., Cooke D.M., Haischer M.H., Carzoli J.P., Johnson T.K., Cross M.R., Cronin J.B., Storey A.G., Zourdos M.C. (2018). RPE vs. percentage 1RM loading in periodized programs matched for sets and repetitions. Front. Physiol..

[44] Keens T.G., Bryan A.C., Levison H., Ianuzzo C.D. (1978). Developmental pattern of muscle fiber types in human ventilatory muscles. J. Appl. Physiol..

[45] Leith D.E., Bradley M. (1976). Ventilatory muscle strength and endurance training. J. Appl. Physiol..

[46] Brannon F.J., Foley M.W., Starr J.A., Saul L.M. (1998). Cardiopulmonary Rehabilitation: Basic Theory & Application.

[47] Gething A.D., Williams M., Davies B. (2004). Inspiratory resistive loading improves cycling capacity: A placebo controlled trial. Br. J. Sports Med..

[48] Hides J., Stanton W., Wilson S., Freke M., McMahon S., Sims K. (2012). Retraining motor control of abdominal muscles among elite cricketers with low back pain. Scand. J. Med. Sci. Sports.

[49] Mohan V., Paungmali A., Sitilertpisan P., Henry L.J., Omar F.A., Azhar F.Z. (2020). The effect of core stability training with ball and balloon exercise on respiratory variables in chronic non-specific low back pain: An experimental study. J. Bodyw. Mov. Ther..

[50] HajGhanbari B., Yamabayashi C., Buna T.R., Coelho J.D., Freedman K.D., Morton T.A., Reid W.D. (2013). Effects of respiratory muscle training on performance in athletes: A systematic review with meta-analyses. J. Strength Cond. Res..

[51] Seixas M.B., Almeida L.B., Trevizan P.F., Martinez D.G., Laterza M.C., Vanderlei L.C.M., Silva L.P. (2020). Effects of Inspiratory Muscle Training in Older Adults. Respir. Care.

[52] Downey A.E., Chenoweth L.M., Townsend D.K., Ranum J.D., Ferguson C.S., Harms C.A. (2007). Effects of inspiratory muscle training on exercise responses in normoxia and hypoxia. Respir. Physiol. Neurobiol..

[53] Condessa R.L., Brauner J.S., Saul A.L., Baptista M., Silva A.C.T., Vieira S.R.R. (2013). Inspiratory muscle training did not accelerate weaning from mechanical ventilation but did improve tidal volume and maximal respiratory pressures: A randomised trial. J. Physiother..

[54] Neves L., Chiappa A., da Silva V., Vieira P., Cipriano G., Arena R., Chiappa G. (2014). Comparative effects of inspiratory muscle training and resistance training on respiratory and skeletal muscle strength in COPD: Responses of the pulmonary rehabilitation program. Eur. Respir. J..

[55] Kraemer W.J., Adams K., Cafarelli E., Dudley G.A., Dooly C., Feigenbaum M.S., Fleck S.J., Franklin B., Fry A.C., Hoffman J.R. (2002). American College of Sports Medicine position stand. Progression models in resistance training for healthy adults. Med. Sci. Sports Exerc..

[56] Morton R.W., Oikawa S.Y., Wavell C.G., Mazara N., McGlory C., Quadrilatero J., Baechler B.L., Baker S.K., Phillips S.M. (2016). Neither load nor systemic hormones determine resistance training-mediated hypertrophy or strength gains in resistance-trained young men. J. Appl. Physiol..

[57] Schoenfeld B.J., Grgic J., Ogborn D., Krieger J.W. (2017). Strength and hypertrophy adaptations between low-vs. high-load resistance training: A systematic review and meta-analysis. J. Strength Cond. Res..

[58] Grgic J., Schoenfeld B.J. (2018). Are the hypertrophic adaptations to high and low-load resistance training muscle fiber type specific?. Front. Physiol..

[59] Hodges P.W., Cresswell A.G., Daggfeldt K., Thorstensson A. (2000). Three dimensional preparatory trunk motion precedes asymmetrical upper limb movement. Gait Posture.

[60] Russell P.J., Phillips S.J. (1989). A preliminary comparison of front and back squat exercises. Res. Q. Exerc. Sport.

[61] Raymakers J.A., Samson M.M., Verhaar H.J.J. (2005). The assessment of body sway and the choice of stability parameter(s). Gait Posture.

[62] Bojairami I.E., Driscoll M. (2022). Coordination between trunk muscles, thoracolumbar fascia, and intra-abdominal pressure toward static spine stability. Spine.

[63] Fan C., Fede C., Gaudreault N., Porzionato A., Macchi V., De Caro R., Stecco C. (2018). Anatomical and functional relationships between external abdominal oblique muscle and posterior layer of thoracolumbar fascia. Clin. Anat..

[64] Vleeming A., Schuenke M. (2019). Form and force closure of the sacroiliac joints. PM R.

[65] Kitamura T., Kido A., Ishida Y., Kobayashi Y., Tsukamoto S., Tanaka Y. (2019). Muscle activity pattern with a shifted center of pressure during the squat exercise. J. Sports Sci. Med..

[66] Braidot A.A., Brusa M.H., Lestussi F.E., Parera G.P. (2007). Biomechanics of front and back squat exercises. J. Phys. Conf. Ser..

[67] Zink A.J., Whiting W.C., Vincent W.J., McLaine A.J. (2001). The effects of a weight belt on trunk and leg muscle activity and joint kinematics during the squat exercise. J. Strength Cond. Res..

[68] Lander J.E., Simonton R.L., Giacobbe J.K. (1990). The effectiveness of weight-belts during the squat exercise. Med. Sci. Sports Exerc..

[69] Gholami-Borujeni B., Yalfani A., Ahmadnezhad L. (2020). Eight-week inspiratory muscle training alters electromyography activity of the ankle muscles during overhead and single-leg squats: A randomized controlled trial. J. Appl. Biomech..

[70] Bazrgari B., Shirazi-Adl A., Arjmand N. (2007). Analysis of squat and stoop dynamic liftings: Muscle forces and internal spinal loads. Eur. Spine J..

